# New products

**DOI:** 10.1155/S1463924600000171

**Published:** 2000


Journal of Automated Methods & Management in Chemistry, Vol. 22, No. 4 (July–August 2000) pp. 115–121

### New products

### Enter the Millenniums

At Pittcon 2000 in New Orleans, USA, PS Analytical exhibited the latest Millennium system for the determination and speciation of mercury and the hydride-forming elements.

PS Analytical's instrumentation is based on the use of atomic fluorescence detection systems primarily coupled to vapour generation techniques. To illustrate the power of this technology, PSA contributed eight poster and two oral presentations to the Technical Conference. These presentations ranged from discussion of the ability of the Millennium range to relate to US EPA standards as well as ISO and CEN related standard methods to newer developments in the field of process chemistry.

Recent experiences with the application of the AFS technology to continuous emission monitors that determine total and ionic mercury levels in stack emissions from coal-fired utilities were also presented. New applications in the petrochemical industry, especially the quantification of low levels (below 1 ppb) in liquid condensates and the speciation profile of mercury in these analytes, were also shown.

Atomic fluorescence spectroscopy coupled to vapour generation or purge-and-trap routines allow detection levels better than 0.1 picograms absolute. PSA displayed its latest technology and also introduced the new process control software and hardware for both liquid and gaseous applications.

### *Why speciate?*

The toxicity, biochemical behaviour and transportation of mercury in the environment is highly dependent on its physio-chemical form. It has been shown that organo-mercury compounds, which may be up to one thousand times more toxic than inorganic mercury, may also be formed through methylation of inorganic mercury by organisms and bacteria, although organo-mercury found in fish and waters is almost always methyl mercury. As with all instrumentation for speciation, it is imperative that the techniques offer good separation and sensitivity in order to quantify the individual species with both accuracy and precision.

Speciation is the determination of the individual physico-chemical forms of an element, which together make up its total concentration in a sample. Knowledge of speciation is important because toxicity, bioavailability, bioaccumulation and transport of a particular element depends critically on the chemical form.

The system involves the coupling of a commercially available HPLC system with a PSA Millennium Merlin. Avalon software is used to control the Millennium Merlin, while data collection is through chromatographic software (or a chart recorder).

One of the main advantages of atomic fluoresence is the sensitivity and linear range it offers. These facts, together with the ease with which it can be coupled to an HPLC system means that it is a simple, reliable and inexpensive method of carrying out mercury speciation.

### *Mercury speciation by HPLC–VG–AFS*

The Millennium Merlin system can be coupled to almost any commercially available HPLC system.

One hundred microlitres of sample solution are injected into a reverse phase column, where an isocratic program is used to separate the species. As the species elute from the column they mix with a stream of acidified bromide/bromate and then pass through a UV reactor. This converts the organomercury species to inorganic mercury, which is then converted to elemental mercury after reacting with $SnCl_2$. The elemental mercury is purged from the gas–liquid separator in a stream of argon, from where it flows through a PermaPure$^{TM}$ membrane to remove any moisture, then on to the detector. Separation and detection of methyl-mercury and ethyl-mercury takes place in less than 10 min.

### *Mercury speciation by GC–AFS*

P S Analytical have developed a fully automated system for gas chromatographic separations linked to the Merlin atomic fluorescence detector. Methodologies have been developed in conjunction with Professor Ron Jones at Florida International University in Miami, USA. The system is built around an Aligent Technologies 6890 (formerly Hewlett Packard) gas chromatograph. Studies on sludge and water samples from the Florida Everglades area have provided an interesting insight in the manner in which mercury enters the food chain.

### *Instrumentation*

A flexible autosampler is provided that allows standard addition multiple sample injection and other features. A unique sample introduction device, the Optic, provides the additional ability to pre-concentrate the samples prior to analysis. The solvent front is eluted prior to transfer to the analytical column. The column efficiency is improved by sample pretreatment and clean-up prior to injection—these precautions avoid column pacification or poisoning. The separated components emerge from the column into the detector area where a conventional detector was replaced by a custom-built pyrolyser linked to the PSA Merlin Atomic Fluoresence Detector. The organo-mercury compounds are directly converted to mercury and transported in a flow of argon carrier gas into the detector housing. The capillary gas flow, the make-up gas flow and the sheath gas flows are optimized to provide minimal peak tailing and to transfer the mercury into the AFS detector for quantization. The analysis output from the AFS detector is fed directly to the A/D converter of commercially available gas chromatography data processing software. The complete instrument can be fully computer controlled to provide precise analytical data.

*Mercury speciation of liquid hydrocarbon samples using gas chromatography–atomic fluorescence spectrometry*

Natural gas and its liquid condensates are primary feedstocks for a variety of industrial processes. The presence of mercury in these samples is not just of environmental concern, but also of economic importance. Heavy financial losses can be incurred by mercury induced corrosion on components used during the production, processing and transportation of natural gas condensates. Aluminium heat exchangers and downstream palladium hydrogeneration catalysts are affected most dramatically.

Mercury removal systems for natural gas have been used effectively for some years and, more recently, removal systems for liquid condensates have become available. The latter application is much more complex given the wide variance in sample matrices. The success of these devices is largely dependent on the physio-chemical forms of mercury in the sample. Capillary gas chromatography coupled to atomic fluorescence spectrometry (GC–AFS) is used to speciate mercury in a range of liquid hydrocarbon samples. Information gained from this approach provides a greater understanding of the distribution of mercury in petroleum applications and enables the optimization of mercury removal strategies.

*Analytical performance*

One of the main advantages of AFS is that the linear range for mercury spans over five orders of magnitude. The absolute detection limit was found to be 0.1 pg. Based on 1 μl injection volume, the method detection limit is around 0.1 ng/ml for liquid hydrocarbon samples. In principle, the programmable temperature vaporization device should allow the injection of larger sample volumes and the method detection limit could be improved. The sensitivity is independent of the chemical species. One further advantage of AFS is its excellent selectivity. Hydrocarbons do not respond to the detector or affect the measurement of mercury. At concentrations ten times higher than the method detection limit, typical precision is around 8% with manual injections. This can be improved to 5% with the use of an autosampler.

The GC–AFS system has been used successfully for mercury speciation in liquid hydrocarbons. Studies to date have proved invaluable for understanding the effectiveness of mercury removal systems. Both quantitative and qualitative data can be obtained. The system is also suitable for elemental mercury, ionic mercury and monoalkyl mercury species. For samples with boiling points greater than 320°C a dilution with an appropriate solvent such as octane is required.

*For further information please contact P S Analytical, Arthur House, Crayfields Industrial Estate, Main Road, Orpington, Kent BR5 3HP, UK. Tel.: + 44 (0) 1689 891211; Fax.: + 44 (0) 1689 896009; e-mail: sales@psanalytical.demon.co.uk; URL—http://www.psanalytical.com*

**FH Niederrhein and Shimadzu develop a complete new analytical system for mercury speciation using HPLC-CVAAS**

Close co-operation between Fachhochsschule Niederrhein (Polytechnic) in Krefeld, Germany, and Shimadzu, has resulted in the development of a new fully integrated analytical system which couples ion-pair chromatography with hydride/cold-vapour-AAS for effective field-use determination of a range of elemental species, e.g. mercury.

Metal speciation via the coupling of elemental spectroscopy and chromatography is considered state-of-the-art methodology in research laboratories, but it is an approach which is hardly used for routine analysis. This is partly because there are so few manufacturers of elemental spectroscopic analytical systems who also manufacture chromatographic systems, making it difficult to find a complete commercial system.

The FH Niederrhein–Shimadzu solution is based on a new species-neutral sample preparation procedure and ion-pair chromatographic system based on RP18 phases using mercaptoethanol as an ion-pairing agent, for the separation of mercury species.

Cold-vapour AAS (CVAAS), based on hydride technology, is an element-specific (for Hg), sensitive and economical analytical method. Individual parameters of the CVAAS have been optimized for the FH Niederrhein–Shimadzu solution, with flow-rates for both systems (CVAAS and HPLC) maximized to yield marked improvements to the determination limit of the CVAAS.

The resulting analytical system is capable of measuring Hg(II) species in the presence of methylmercurychloride in a concentration of 5 μg/1 in aqueous systems. It also retains all of the benefits of Shimadzu chromatography software.

'Elemental speciation is an important issue in the description and evaluation of complex ecological systems', said Peter Sidhu of Shimadzu. 'But it has been recognized for some time that information on the content, for example of heavy metals, is not sufficient for the description of ecological and ecotoxicological parameters such as mobility, enrichment, and the possible effects of these elements on bio- and geochemical metabolic pathways. This new collaborative system is the answer for field-based analysis'.

Because the new FH Niederrhein–Shimadzu product is such a recent development and arose from a research project at the Polytechnic, it is not yet available as a commercial product from Shimadzu. More details will be available at the time of release.

*For more information please contact Ross Heaven, Strong Words, 32 Cranstoun Street, Northampton NN1 3BH, UK. Tel./Fax: (+ 44) (0) 1604 250221; e-mail: rossheaven@aol.com, or Stephanie Parish, Shimadzu Europa (UK Branch), Mill Court,*

*Featherstone road, Wolverton Mill South, Milton Keynes MK12 5RE, UK. Tel.: (+44) (0) 1908 552200; Fax: (+44) (0) 1908 552211; e-mail: Sales@Shimadzu.co.uk*

**ERO to market complete family of temperature controllers in the UK**

ERO Electronic, the well-established Italian manufacturer of temperature controllers and ancillary products, has set up a British operation to market its products in the UK. ERO UK will give direct access to comprehensive ERO product family, including process and temperature controllers, indicators and programmers. A number of specialist distributors will be appointed to provide a comprehensive and expanding sales and support network.

ERO Electronic is now part of the British-owned Invensys Group and a sister company to Eurotherm Controls, Europe's largest manufacturer of temperature controllers. It will base its new ERO UK headquarters in Worthing, West Sussex. Both ERO and Eurotherm Controls are, however, keen to emphasize that their respective product ranges will be positioned to address different market sectors. This complementary positioning will provide the group with wider product coverage and increased overall market share.

Anthony Johns, who was previously Sales Manager of Arcom Controls Systems, is heading up ERO UK. He comments: 'The ERO product range offers a superb choice to both OEMs and control systems users alike. This is clearly illustrated by the high level of acceptance and recognition we already enjoy world-wide. ERO instruments have gained a well-deserved reputation for delivering excellent performance levels and specifications, at a modest price'.

The company has been a major manufacturer of industrial instrumentation and control products for more than 20 years, and is one of the leading Italian companies in this field. With development and manufacturing facilities near Milan, it is an ISO9001 quality organization which invests a significant proportion of its annual revenue in research and development. This has allowed it to create a broad family of state-of-the-art products and systems over time, with a constant flow of new products being added. This continues, and ERO UK will announce several advanced new process control products before the end of the year.

With an already established and growing customer base in this country, the investment in its new Worthing facility is intended to provide ERO with the necessary presence to consolidate UK market successes. In addition, Hawco (based in Guildford) and ThermoSpeed (based in Manchester) will continue to provide excellent distribution and catalogue sales support. Additional specialist distributors will be appointed in the future to develop the UK sales network further.

Anthony Johns believes that ERO's arrival will present a significant challenge to established suppliers in the UK market. He says: 'We shall be offering a number of innovative, technically excellent, but economically priced solutions to control system designers and builders during the coming months. That means a wider choice, better quality standards and increased competition. I would expect that some of our major competitors will get a little uncomfortable as a result, but customers, however, can only benefit'.

*For further information please contact: Anthony Johns, ERO UK, Unit 1, Cygnet Trading Estate, Faraday Close, Durrington, Worthing, W. Sussex BN13 3RQ, UK. Tel.: 01903 693322, Fax: 01903 693377: e-mail: danbogard@cix.co.uk*

**Shimadzu TOC/N on-line analyser for sugar and paper production**

The Shimadzu TOCN-4100 on-line analyser measures total organic carbon (TOC) and total nitrogen content (TN), which are important control parameters for effluent treatment.

The measurement principle involves oxidation at 680°C using a platinum catalyst. Carbon compounds are converted to carbon dioxide and measured using a non-dispersive infra-red detector, while nitrogen compounds are converted to nitric oxide and measured by means of a chemiluminescence detector.

The instrument is supplied with a sampling system which has been designed specifically for applications where high levels of solids are present. The system includes a high-speed rotating mesh filter which is backflushed between samples, and an homogenizing unit which breaks down particulates prior to introduction to the analyser. This system has been successfully employed in a range of industries, e.g. in the processing of sugar and the manufacture of paper, where the presence of large particles can cause severe challenges in sampling.

TOC and TN can be measured from a single sample injection with an analysis time of ~5 min. The results from up to six sample streams may be transmitted via analogue signals or a serial link for displaying trends and alarm generation; automatic calibration and remote operation are all available in the standard unit.

*For more information, please contact Ross Heaven, Strong Words, 32 Cranstoun Street, Northampton NN1 3BH, UK. Tel./Fax: (+44) (0) 1604 25022; e-mail: rossheaven@aol.com or Stephanie Parish, Shimadzu Europa (UK Branch), Mill Court, Featherstone Road, Wolverton Mill South, Milton Keynes MK12 5RE, UK. Tel.: (+44) (0) 1908 552200; Fax: (+44) (0) 1908 552211; e-mail: Sales@Shimadzu.co.uk*

**Syringeless filters cut sample preparation time by 66%. New system increases throughput and reduces costs**

By simply switching to the new Whatman Mini-UniPrep syringeless filter, laboratories can remove particulates from samples in one-third of the time that it currently takes with syringe-coupled devices and autosampler vials.

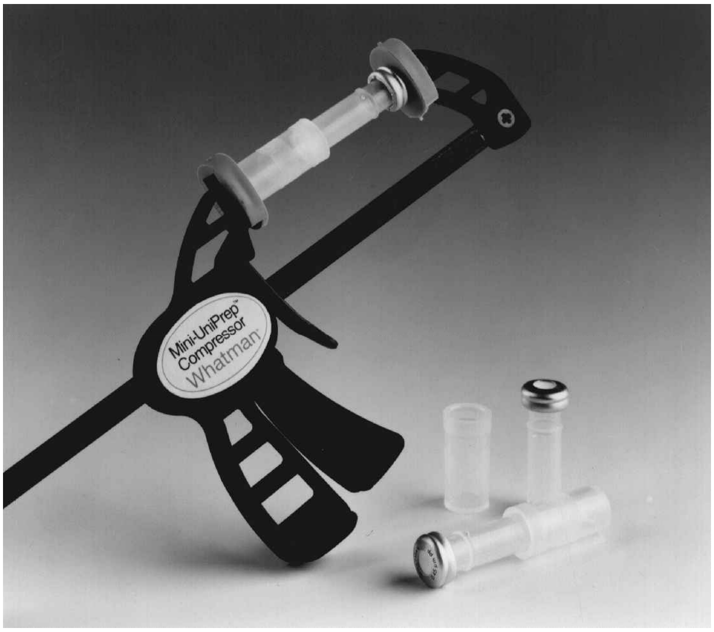

*The new Mini-UniPrep syringeless filter from Whatman. Issued on behalf of Whatman by HCC.De Factor.*

This is because the new system combines the attributes of both into one simple-to-use, disposal unit.

After sample preparation, the Mini-UniPrep can be placed into most autosamplers designed to accommodate 12 × 32 mm vials.

This new approach generates less waste, improves sample throughput without increasing staffing levels, and cuts overall operating costs.

Each UniPrep consists of two parts: a chamber and a plunger. A filtration membrane is located at one end of the chamber and a pre-attached cap/septum at the other. By pressing the plunger through the liquid in the chamber, positive pressure forces the filtrate up and into the reservoir of the plunger. Air escapes through the vent hole until the locking ring is engaged and forms a liquid-tight seal.

Mini-UniPrep syringeless filters are supplied with a 0.45 μm polypropylene filter and are available in packs of 100.

*For further information contact: Kevin Payne, Kelly Newvell, HCC.De Facto. Tel: 01256 842274; Whatman, Tel.: 01622 624239*

### Ultrasonic sensors can now be programmed in seconds, even in awkward locations

*Plug-in tool means engineers can set thresholds remotely at the push of a button*

ABB Control's new remote programming tool virtually eliminates the problems of setting up ultrasonic position sensors—traditionally an awkward job. Ultrasonic sensors have near-immunity to dirty conditions and a long sensing range, but they can be difficult to set up because the instrumentation engineer must physically reach the sensor to set the thresholds. In many installations this is difficult or impossible.

The new programming tool simply connects via a 1.5-m cable between the sensor and its connector cable; all that is needed are two button pushes to set maximum and minimum sensing values, then the unit can be removed. This solution is more accurate than the alternative of setting thresholds via potentiometers, which can 'drift'. ABB Control expects that the new tool will lead to increased use of sensors of this type.

Conrad Slater, ABB Control's sensor specialist, expects that the new tool will lead to increased use of ultrasonic sensors: 'They have many benefits', he says. 'They can be used to detect position from any sound-reflective surface at a distance up to 6 m—much greater than capacitive and inductive sensors—and unlike optical sensors, they are self-cleaning and immune to dust, so they can operate in harsh conditions'.

Ultrasonic sensors determine position by measuring the time taken for an ultrasonic pulse to be reflected from the substrate. Output responds to changes in position typically within 100 ms, and are linear to within 0.1%. Resolution can be better than 0.2 mm even at a sensing distance of several metres.

The outputs of ABB Control's ultrasonic sensors are automatically compensated for changes in ambient temperature. Shock and vibration performance exceed the EN 60974-5-2 standard, and the sensors have IP65 ingress protection rating.

ABB Control now has a range of 200 position sensors, sufficient to meet 80% of system builder, OEM and end-user needs. They are available from ABB Control's Coventry distribution centre and from a network of distributors.

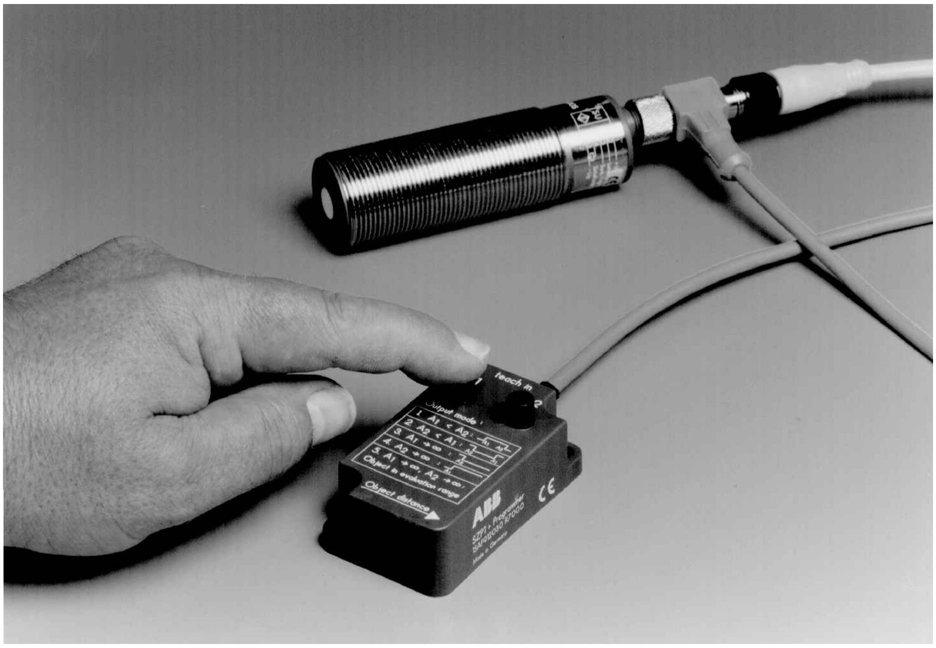

*ABB Control's new remote programming plug-in tool.*

*For further information please contact: Conrad Slater, ABB Control. Tel.: (01203) 368500; Fax: (01203) 368401. Danny Dicks, Roger Staton Associates, Tel.: (01628) 487222; Fax: (01628) 487223*

### Quality in Autosampling from Bio-Tek

Bio-Tek Instruments, based in Watford, has established a strong presence in the field of analytical instrumentation. The latest development is the 565 variable injection autosampler, designed to satisfy the most demanding requirements.

Part of the HPLC 500 system, the 565 Autosampler is ideal for high-throughput applications, having a range of variable volumes to be injected. From microbore to analytical, the 565 will ensure full optimization of your injections.

Built on a proven design, the 565 Autosampler offers unmatched reliability, allowing you to perform unattended and prolonged analyses with total peace of mind. With all features accessible through an easy-to-use software program, the 565 Autosampler is another example of Bio-Tek's commitment to quality.

*For further information, please contact: Colin Fallowfield, Biotek Instruments, 8 Marlin House, Croxley Buisness Park, Watford, Hertfordshire WD1 8YA, UK, Tel.: + 44 (0) 1923 691300*

### Solvotrode—the ideal electrode for non–aqueous acid–base titrations

The new 6.0229.100 Metrosensor Solvotrode with individual quality certificate is a combined pH glass electrode which has been specially developed for non-aqueous acid–base titrations. It can be used in many cases where it was previously necessary to use complicated set-ups with two or even three electrodes. Non-aqueous titrations play an important role in quality control in the pharmaceutical industry, oil industry, and in plastics and foodstuffs (fats/oils).

The pH glass membrane of the Solvotrode is made of low- impedance glass and forms a relatively large sphere. This means that stable measurements are achieved even in poorly conducting solutions, e.g. non-aqueous media.

The built-in reference electrode naturally contains the well-proven 'long-life' reference system with Ag/AgCl cartridge and diffusion barrier for $Ag^+$ ions. The Solvotrode is equipped with a PCTFE (polychlorotrifluoroethylene) ground-joint diaphragm that is both easy to clean and less sensitive to contamination/blockage.

The Solvotrode is filled with saturated LiC1 in ethanol as standard and is immediately ready for use. However, for titrations with KOH or TBAOH it is recommended to use tetraethylammonium bromide (TEABr) in ethylene glycol (order no. 6.2320.000) as the reference electrolyte. The inner tube of the Solvotrode is coated with a special conductor which, independent of the electrolyte level, ensures that the set-up is optimally sheilded. This additional sheilding is particularly important for measurements in non-aqueous media as it virtually eliminates interferences due to static electricity.

*For further information please contact: Metrohm UK , Unit 2, Top Angel, Buckingham Industrial Park, Buckinghamshire, MK18 1TH, UK. Tel.: + 44 (0) 1280 824824*

### Low-level moisture analysis

The new 756 KF Coulometer from Metrohm leaves no wish unfulfilled for analysts who wish to determine moisture levels down to 1 part per million. Continuing Metrohm's successful and established line of Karl Fischer instruments, this latest addition guarantees accurate and reproducible results.

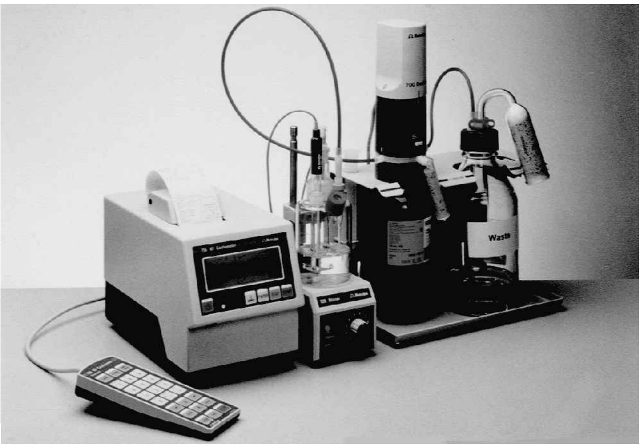

*The new 756 KF Coulometer from Metrohm.*

The 756 has a large clear backlit screen which not only provides clear presentation of parameters, but also shows graphically the course of the KF titration as it progresses. This facilitates the integrity of each determination as well as providing valuable information for method development, e.g. differentiation between surface and water of crystallitaztion.

The 756 has two RS232 interfaces enabling PC, printer or balance connection as well as the possibility to connect a bar code scanner or PC keyboard.

For samples where the moisture content is totally unknown, a variable current generator comes into its own, generating large amounts of KF reagent for high moistures and small amounts of µg range. GLP (good laboratory practice) functions, e.g. results specification, min/max permissible weights, service/calibration dates, may be programmed in.

The 756 comes with a choice of generating electrodes: without diaphragm for trouble-free one-reagent analysis; or the twin reagent cell for ketone and aldehyde analysis.

The 756 can monitor the reagent consumed at all times, thus it is possible to program into the instrument to change the regent when either a certain time has elapsed (e.g. 7 days) or more likely when a certain amount of $H_2O$ has been titrated (e.g. 800 mg $H_2O$).

By connecting a 700 Dosino, it is also possible to change this reagent automatically without the cell being opened and consequently allowing moisture into the cell. Different applications demand different parameters; to facilitate this the 756 can permanently store over 100 methods and may also be connected to a PC for additional memory.

*For further information please contact: Metrohm UK. Tel.: + 44 (0) 1280 824 824*

### Automate content uniformity and composite assays

The Zymark Tablet Processing Workstation II (TPW II) is a benchtop instrument designed to automate sample preparation for solid dosage forms. Tablets, capsules, powders, blends and medicated feeds have been successfully quantitatively analysed by the TPW II.

Samples that previously required hours to dissolve by shaking or sonicating are extracted in seconds using an innovative wet grinding homogenization technique that produces reliable and reproducible results. Extracted samples can be filtered and diluted, with the final sample solution being directly injected onto an HPLC, read via on-line UV/VIS spectroscopy, or stored in test tubes or vials. A clear and complete record of every sample preparation step is provided. Two electronic balances monitor solvent dispensing, liquid handling and sample handling operations. This information is recorded in an electronic audit trail.

The TPW II reduces typical sample preparation times and increases sample throughput. Laboratory productiv-

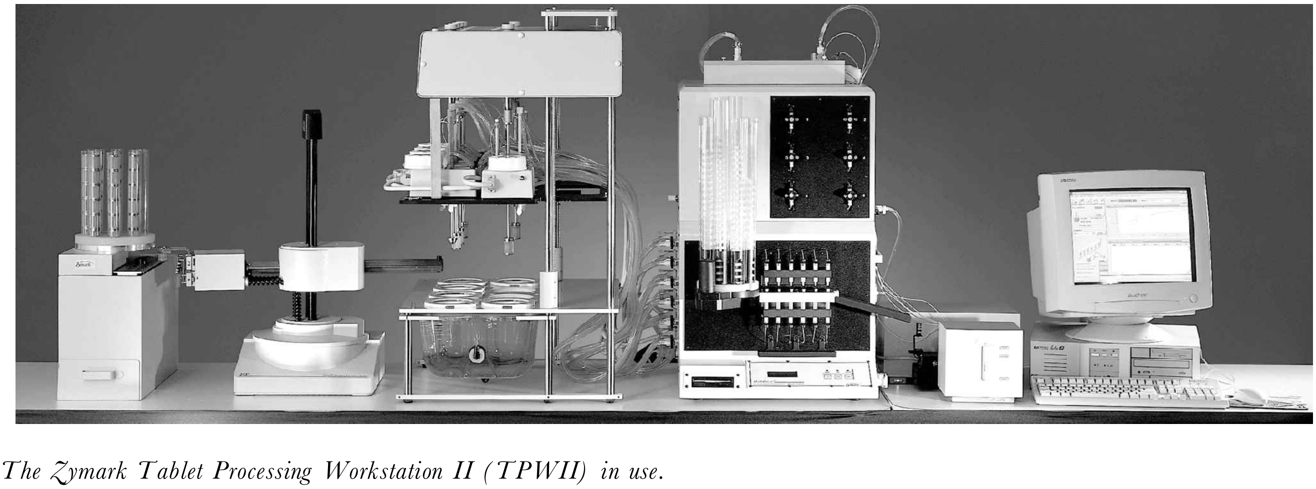

*The Zymark Tablet Processing Workstation II (TPWII) in use.*

ity can be significantly increased by using the TPW II for extended hours of operation, e.g. overnights and over weekends. Up to 100 samples can be staged for content uniformity or composite assay analysis using any combination of up to 10 different methods without human intervention.

Zymark impacts the world by advancing the ability to discover, develop and market safe and effective drugs that can lengthen and improve the quality of life for everyone. Zymark's employees are active in more than 35 countries and their combined expertize in pharmaceutical analysis and laboratory automation makes Zymark the world's leading supplier in this application area.

*For more information contact: Sharon Correia, Director of Marketing Communications, Zymark Corporation, Zymark Center, Hopkinton, MA 01748, USA. Tel.: + 1 (508) 497-6403. e-mail: sharon.correia@zymark.com*

### A new definition to detection

Bio-Tek Instruments have long been at the forefront of technology in analytical instrumentation. One of the latest developments is a new line of HPLC diode array detectors for detection in the UV and UV–Vis range. The DAD540+ and DAD 545 feature PeakMax™ fibre optics technology, a Bio-Tek breakthrough.

Fibre optics has been proven to be the most efficient way to transfer the energy emitted by the lamps to the flow cell and from the cell to the polychromator. Light is picked up in the region where the energy density is highest. The small image of the source is then focused in three types of high-efficiency flow cells and modulated onto the array of diodes. The results are outstanding in both chromatography and spectroscopy.

The range of available analytical flow cells covers all HPLC applications from analytical to microbore, from high-speed to high-resolution and trace analysis. Long fibre optics allow the flow cell to be positioned outside the detector module without the risk of deterioration of chromatographic and spectral performance. For total reliability in identity and purity information, the 540+ and 545 diode array detectors redefine the limits of modern technology.

*For further information please contact: Colin Fallowfield, Bio-Tek Instruments, 8 Marlin House, Croxley Business Park, Watford, Hertfordshire WD1 8YA, UK. Tel.: + 44 (0) 1923 691300*

### Column Thermostat from Bio-Tek—Sophistication in HPLC

Bio-Tek Instruments, based in Watford, is one of the world's leading manufacturers and suppliers of instrumentation for analytical chemistry. Bio-Tek products feature the very latest technology for optimum performance in any situation. As a result of continuing research and development, the company has recently introduced the 582 Column Thermostat.

A part of the best-selling HPLC 500 system, the Column Thermostat is a stackable module. This means that the capillary connection length from the autosampler to column and detector is minimized. With modern applications requiring stable temperatures to achieve and keep the proper column environment for optimum separation conditions, these shorter connections mean superior resolution, and reproducibility are ensured at all times.

The 582 Column Thermostat uses a state-of-the art Peltier system to attain rapid heating/cooling speeds for good retention time stability. From routine application to the most advanced research requirements, the 582 Column Thermostat offers total accuracy and reliability.

*For further information please contact: Colin Fallowfield, Bio-Tek Instruments, 8 Marlin House, Croxley Business Park, Watford, Hertfordshire WD1 8YA, UK. Tel.: + 44 (0) 1923 691300*

### Environmental monitoring of plants with the SIMAA 6000

An application note, available from Perkin-Elmer, discusses the determination of lead, cadmium, chromium, nickel, and copper in plants to monitor the effects of environmental pollution. The SIMAA 6000 simultaneous atomic absorption spectrometer with transversely heated graphite furnace and Zeeman-effect background correction was used to analyse samples prepared by a multiwave microwave digestion system.

The methodology and results presented demonstrate the potential of simultaneous graphite furnace AAS for the accurate determination of trace metals in plants. In addition, significant timesavings result from the use of fast microwave sample digestion, followed by the simultaneous atomic absorption determination of five elements.

*For further information, please contact: Carol-Anne Green, Perkin-Elmer UK, Post Office Lane, Beaconsfield, Buckinghamshire HP9 1QA, UK. Tel.: + 44 (0) 1494 679269; Fax: + 44 (0) 1494 877025; e-mail: greenca@eur.perkin-elmer.com*

